# Supplementary material for: Environmental fluctuations alter the competitive trade-offs of group size in a social primate
Source: Nat Ecol Evol. 2026 May 6;10(5):919–31. doi: 10.1038/s41559-026-03048-8 (PMC13167455; doi:10.1038/s41559-026-03048-8)
Supplement: Supplementary file 2 — Reporting Summary [file 41559_2026_3048_MOESM2_ESM.pdf]

nature portfolio

Corresponding author(s): Odd T. Jacobson

Last updated by author(s): Mar 6, 2026

Reporting Summary

Nature Portfolio wishes to improve the reproducibility of the work that we publish. This form provides structure for consistency and transparency in reporting. For further information on Nature Portfolio policies, see our Editorial Policies and the Editorial Policy Checklist.

Statistics

For all statistical analyses, confirm that the following items are present in the figure legend, table legend, main text, or Methods section.

n/a	Confirmed	
☐	☒	The exact sample size (n) for each experimental group/condition, given as a discrete number and unit of measurement
☐	☒	A statement on whether measurements were taken from distinct samples or whether the same sample was measured repeatedly
☒	☐	The statistical test(s) used AND whether they are one- or two-sided<br>*Only common tests should be described solely by name; describe more complex techniques in the Methods section.*
☐	☒	A description of all covariates tested
☐	☒	A description of any assumptions or corrections, such as tests of normality and adjustment for multiple comparisons
☐	☒	A full description of the statistical parameters including central tendency (e.g. means) or other basic estimates (e.g. regression coefficient) AND variation (e.g. standard deviation) or associated estimates of uncertainty (e.g. confidence intervals)
☒	☐	For null hypothesis testing, the test statistic (e.g. F, t, r) with confidence intervals, effect sizes, degrees of freedom and P value noted<br>*Give P values as exact values whenever suitable.*
☐	☒	For Bayesian analysis, information on the choice of priors and Markov chain Monte Carlo settings
☐	☒	For hierarchical and complex designs, identification of the appropriate level for tests and full reporting of outcomes
☐	☒	Estimates of effect sizes (e.g. Cohen's d, Pearson's r), indicating how they were calculated

Our web collection on statistics for biologists contains articles on many of the points above.

Software and code

Policy information about availability of computer code

Data collection	Observational data were recorded in the field using Terminal Emulator for Android on handheld computers. Movement data were collected with Garmin GPSMap devices (62s, 64, 64s, 66sr) and organized using Garmin BaseCamp (version 4.7.5). Environmental data were obtained via the Google Earth Engine Python API (Spyder 6.0.7, Python 3.11.12, Qt 5.15.15, PyQt5 5.15.11, Windows 10).
Data analysis	All analyses were conducted in R (version 4.5.0) using RStudio Server (2024.12.1+563).

For manuscripts utilizing custom algorithms or software that are central to the research but not yet described in published literature, software must be made available to editors and reviewers. We strongly encourage code deposition in a community repository (e.g. GitHub). See the Nature Portfolio guidelines for submitting code & software for further information.

Data

Policy information about availability of data

All manuscripts must include a data availability statement. This statement should provide the following information, where applicable:

- Accession codes, unique identifiers, or web links for publicly available datasets
- A description of any restrictions on data availability
- For clinical datasets or third party data, please ensure that the statement adheres to our policy

Location data are restricted to protect the precise whereabouts of habituated, threatened primates vulnerable to the pet trade. They are archived in a limited-access Movebank repository here: https://www.movebank.org/cms/webapp?gwt_fragment=page=studies,path=study6448057425. These can be made available

upon reasonable request to Prof. Susan Perry. All code necessary to reproduce our analyses are openly available on Edmond here: https://doi.org/10.17617/3.GBGJCM. All demographic and behavioral data are openly available on Dryad here: https://doi.org/10.5061/dryad.612jm64j0.

Research involving human participants, their data, or biological material

Policy information about studies with human participants or human data. See also policy information about sex, gender (identity/presentation), and sexual orientation and race, ethnicity and racism.

Reporting on sex and gender	N/A
Reporting on race, ethnicity, or other socially relevant groupings	N/A
Population characteristics	N/A
Recruitment	N/A
Ethics oversight	N/A

Note that full information on the approval of the study protocol must also be provided in the manuscript.

Field-specific reporting

Please select the one below that is the best fit for your research. If you are not sure, read the appropriate sections before making your selection.

☐ Life sciences ☐ Behavioural & social sciences ☒ Ecological, evolutionary & environmental sciences

For a reference copy of the document with all sections, see nature.com/documents/nr-reporting-summary-flat.pdf

Ecological, evolutionary & environmental sciences study design

All studies must disclose on these points even when the disclosure is negative.

Study description

Aim: This study examines how environmental fluctuations alter the balance between within- and between-group competition, and how these shifts shape group behavior and intergroup interactions at the population scale.

Study system and data: The analysis is based on 33 years (1991–2023) of behavioral and spatial observations from 12 wild groups of white-faced capuchins (Cebus imitator) monitored by the Lomas Barbudal Monkey Project, Costa Rica, since 1990.

Study design structure: Observational, repeated-measures, hierarchical/multilevel design with (i) group-, individual-, and group-dyad-level effects and (ii) nested time scales (daily, seasonal [dry/wet], annual). Dyadic analyses use a hierarchical social-relations framework (focal, neighbor and group-dyad effects).

Predictors ("treatment" factors) and interactions: (i) group size, (ii) season (dry vs. wet), (iii) vegetation productivity (NDVI), (iv) Standardized Precipitation–Evapotranspiration Index (SPEI), (v) dyadic group size (interaction between focal and neighbor group size). Other interactions include: group size x season (group-level models), group size x season x SPEI (fruit intake and range NDVI group-level models), focal group size x neighbor group size x season (dyadic models).

Responses: (i) Within-group models: (a) per-capita fruit ingestion rate, (b) daily path length, (c) return/revisitation rate, (d) home-range area, (e) home-range quality. (ii) Between-group models: (f) home-range overlap and (g) intergroup encounter rate.

Experimental units and replication: (i) Individual-day (fruit ingestion): daily observations nested within 335 individuals nested within 12 groups (varying intercepts/slopes for individuals and groups). (ii) Group-day (daily path length): 996 daily observations nested within 12 groups (varying intercepts/slopes by group). (iii) Group-year and group-season-year (home-range area/quality, revisitation): repeated measures per group across 33 years and 66 seasons/years. (iv) Dyad-year and dyad-season-year (overlap, encounter rate): repeated measures for group pairs; up to 66 possible dyads across 12 groups.

Research sample

The research sample comprises 33 years (1991–2023) of behavioral and spatial observations from 12 wild, habituated groups of white-faced capuchins (Cebus imitator) monitored by the Lomas Barbudal Monkey Project in Costa Rica. We aimed to follow as many groups as feasible while maintaining sufficient detail per group to capture between-group variation and population-level patterns. White-faced capuchins are platyrrhine primates well known for their advanced cognitive capacity, large brain-to-body size ratios, omnivorous diets, extractive foraging, cooperation, coalitionary behavior, long life spans, and slow life histories. They live in cohesive multi-male, multi-female groups of roughly 5–40 individuals (mean ≈ 18.8), spanning ages from 1 month to 34 years in our dataset. The sample is intended to represent the free-ranging white-faced capuchin population inhabiting the tropical dry-forest ecosystem surrounding the Lomas Barbudal Biological Reserve and comparable habitats across the Americas. More broadly, the findings have relevance for understanding between-group conflict, resource partitioning, and other ecological trade-offs faced by social animals across taxa, including humans.

We analyze existing datasets compiled by the project. Behavioral data include focal follows collected between July 2006 and June

2021, yielding 4,952 hours of usable observations from 335 individually identified capuchins. Spatial data come from handheld GPS devices carried by observers following habituated groups between September 2009 and April 2020, supplemented by historical location records extracted from field notes spanning 1990–2023 (e.g., sleep sites and positions at trail crossings and landmarks). Environmental data were derived from openly available satellite products, including surface reflectance from Landsat 5/7/8 (via Google Earth Engine) and tree cover from the Hansen Global Forest Change dataset. Climate data was retrieved from the ERA5 Climate Reanalysis dataset.

Sampling strategy

We analyzed the full longitudinal record available from the Lomas Barbudal Monkey Project, using all years, groups, and individuals that met predefined data-quality criteria. This choice reflects the study's observational nature and our aim to capture the widest feasible range of environmental conditions, demographic variation, and intergroup relationships. Sampling intensity varied depending on funding and staff availability, meaning that only one or two groups could usually be monitored at a time. The choice of which group to follow on a given day depended on ongoing project priorities and on which individuals were most in need of updating their focal follow hours for that month. Behavioral focal follows were collected under established protocols for longitudinal studies involving habituated diurnal animals (Perry 2012; https://doi.org/10.1016/B978-0-12-394288-3.00004-6) and we applied basic quality control measures (e.g., excluding incomplete days and spurious locations) where appropriate.

For metrics derived from continuous-time movement models, sample sufficiency was assessed via diagnostics that measure effective information content rather than raw point counts (Fleming et al. 2019; https://doi.org/10.1111/2041-210X.13270). These diagnostics test whether a data segment (day, season, or year, depending on the analysis) contained enough independent information (typically about ten home-range crossings) to estimate parameters such as home-range size, revisitation, or overlap. Segments that failed range-residency tests or fell below this effective sample size threshold were excluded. We further checked that retained data were temporally representative within each analysis window (Jacobson et al. 2024; https://doi.org/10.1007/s10764-023-00398-z).

For statistical models, we relied on Bayesian generalized linear mixed models that make full use of the study's hierarchical and repeated-measures structure. These models included varying intercepts and slopes at the individual, group, and dyad levels, enabling partial pooling. In practice, this means that when some groups or individuals had fewer observations, their estimates were stabilized by borrowing information from the broader dataset, avoiding biased or unreliable results due to uneven sampling. Model adequacy was confirmed through posterior predictive checks, and precision was ensured by the large number of repeated observations across daily, seasonal, and annual scales combined with the breadth of demographic and environmental variation in the dataset.

Data collection

Capuchin groups were followed daily from dawn to dusk (typically 05:00–18:15), as they moved from one sleeping site to the next. Because capuchins show little nocturnal activity, observers left groups at nightfall and returned before first light. At each morning sleep site, observers enabled the tracking function on a handheld Garmin GPS unit, which logged locations at ~30-s intervals until the group settled at its evening tree. On days when groups were first encountered in the forest ("search days"), GPS tracking began upon contact and continued until the group reached its sleeping site. Tracking was occasionally terminated early if groups were lost under difficult conditions or if observers alternated between study groups.

While following the monkeys, trained observers collected behavioral data through focal follows, usually 10 minutes in duration (though sometimes longer depending on concurrent projects). During these follows, every behavior of the focal individual was recorded, including foraging and food-processing actions and social interactions. Between focal follows, group scans were conducted to provide snapshots of visible individuals, their activities, and proximity to other group members. Daily censuses were also completed, documenting group membership and health and reproductive status. All individuals were recognized by experienced observers through morphological characteristics.

Before the introduction of handheld GPS units in late 2009, spatial data (especially sleep sites) were documented in field notebooks or handheld Psion devices. Observers described locations relative to salient landmarks such as rivers, trails, waterfalls, and cliffs. These records were essential for relocating groups each morning and maintaining continuity in long-term behavioral sampling prior to the adoption of GPS technology (see Jacobson et al. 2024; https://doi.org/10.1111/ele.14443).

In addition to Susan Perry, Odd Jacobson, and Brendan Barrett, the following field assistants contributed with data collection: J. Anderson, C. Angyal, L. Appleby, K. Atkins, A. Autor C., M. Bergstrom, R. Berl, L. Beaudrot, T. Bishop, A. Bjorkmann, L. Blankenship, T. Borcuch, J. Broesch, A. Büry, D. Bush, J. Butler, F. Campos, C. Carlson, S. Carnegie, S. Caro, L. Chuaqui, A. Cobden, C. Collins, G. Corradini, M. Corrales, J. Damm, B.A. Davis, C. deRango, C. Dillis, N. Donati, G. Dower, R. Dower, A. Duchesneau, K. Feilen, J. Fenton, S. Fiello, K. Fisher, A. Fuentes J., M. Fuentes, T. Fuentes A., A. Gaston, C. Gault, H. Gilkenson, M. Glenwright, I. Godoy, I. Gottlieb, J. Griciute, J. Gros-Louis, L.M. Guevara R., M. Guimond, L. Hack, M. Hammel, R. Hammond, R. Hamrick, A. Hanadari-Levy, S. Herbert, C. Hirsch, M. Hoffman, A. Hofner, C. Holman, J. Hubbard, S. Hyde, M. Jackson, S. Jackson, E. Johnson, K. Kajokaite, M. Kay, E. Kennedy, D. Kerhoas-Essens, S. Kessler, D. Khieninson, P. Kolence, W. Krimmel, W. Lammers, M. Lechner, S. Lee, S. Leinwand, L. Johnson, S. Lopez Plaza, T. Lord, S. MacCarter, J. Mackenzie, F. McKibben, J. Manson, M. Mayer, W. Meno, A. Mensing, M. Milstein, A. Mitchell, C. Mitchell, W. Meno, J. Mudde, Y. Namba, D. Negru, A. Neyer, C. O'Connell, J.C. Ordoñez J., F. Ouweleen, N. Parker, B. Pav, S. Pereira, K. Perry, J. Pinnock, R. Popa, K. Potter, K. Ratliff, K. Reinhardt, N. Roberts B., E. Rothwell, J. Rottman, H. Ruffler, S. Sanford, C.M. Saul, S. Schading, I. Schamberg, S. Schembari, N. Schleissmann, K. Schleper, C. Schmitt, S. Schulze, A. Scott, E. Seabright, J. Shih, L. Sirot, S. Sita, M. Skuja, J. Stampfl, K. Stewart, W.C. Tucker, E. Urquhart, J. Vandermeer, K. van Atta, L. van Zuidam, J. Verge, G. Viallon, V. Vonau, R. Wakeford, A. Walker-Bolton, K. Watz, E. Wikberg, M. White, E. Williams, J. Williams, E. Wolf, D. Wood, D. Works, and M. Ziegler. Long-term site managers H. Gilkenson and W. Lammers made particularly large contributions.

Timing and spatial scale

Data collection for this study spans from 18 May 1991 to 24 September 2023. Because field staff were limited and groups were followed on a rotating basis, sampling effort was uneven across groups and years, leading to data gaps for particular groups. These gaps and their implications for spatial analyses were evaluated in detail by Jacobson et al. (2023; https://doi.org/10.1007/s10764-023-00398-z). That analysis demonstrated that missing data did not bias home-range estimates provided that observations were distributed across at least 5–10 unique weeks within a given season or year. Following those results, we included only data segments that met or exceeded these thresholds.

Sampling frequency and periodicity varied: groups were observed for runs of 1–22 consecutive days, with focal follows and GPS

	tracking conducted continuously during daylight hours. Exact sampling periods for each group are shown in Supplementary Figure S1. The spatial scale of the study site encompasses approximately 10 km² of tropical dry forest within and adjacent to the Lomas Barbudal Biological Reserve, Costa Rica, which includes the overlapping home ranges of all monitored groups.
Data exclusions	Data segments were excluded if they did not meet pre-established thresholds for sample size sufficiency. Most notably, data from 1990 were too sparse and therefore excluded, and one group (DT) was removed due to insufficient location data; thus, although 13 groups were habituated, only 12 are included in the analyses. In addition, GPS tracking data collected after April 2020 were not yet fully cleaned and processed, so only GPS data from September 2009–April 2020 were included in the analyses. Sleep-site data, however, span continuously from 1991 through September 2023. All exclusions were based on predefined criteria established in previous validation work (Jacobson et al. 2023; Jacobson et al. 2024).
Reproducibility	Our study is observational and does not involve experiments, but all analyses are fully reproducible. Code, workflows, and documentation are openly available at https://doi.org/10.17617/3.GBGJCM, with detailed annotations. No attempts at replication have failed; the analyses can be rerun to yield the same results.
Randomization	Our study did not involve randomized experiments, as it is based on long-term observational data. Instead, we controlled for covariates statistically using a causal inference framework. For each model, we developed directed acyclic graphs (DAGs) to make our causal assumptions explicit and to guide covariate selection. The rationale and details of this approach are provided in Section S1.4 of the Supplementary Material.
Blinding	Blinding was not relevant to this study because it was purely observational (no treatment groups). All groups of capuchins are natural social units that exist in the wild, and all individuals are identified by trained observers. Rigorous training and regular examinations ensured that all data was collected reliably and to the highest standard.

Did the study involve field work? ☒ Yes ☐ No

Field work, collection and transport

Field conditions	The study was conducted in tropical dry forest in Guanacaste, Costa Rica, within and around the Lomas Barbudal Biological Reserve. The climate is strongly seasonal, with a dry season (December–April) and a wet season (May–November). During the dry season, daily maximum temperatures average 30–35 °C with near-zero rainfall, while in the wet season temperatures average 27–32 °C and daily precipitation ranges from ~5 to >300 mm (Figure 1; ERA5). The terrain is moderately hilly and includes continuous forest inside the reserve as well as fragmented pasture–forest mosaics with riparian corridors outside. Rivers are a regular feature of the landscape and were often crossed either while following monkeys or when traveling between sleeping sites and vehicles. The reserve is also notable for high bee and wasp diversity and the presence of venomous snakes (e.g., rattlesnakes); field teams followed standard safety protocols, including avoiding wearing snake leggings and maintaining contact with park rangers. For much of the study, observers resided in a nearby town (~30 min by vehicle) and commuted daily, arriving before dawn and returning after dusk; at other times they lived inside the reserve and walked directly to sleep sites.
Location	Within and nearby the Lomas Barbudal Biological Reserve in Guanacaste, Costa Rica (10∘29–32’N, 85∘21–24’W). The elevation is around 10-180m (Frankie et al. 1993; https://doi.org/10.2307/2388320).
Access & import/export	We obtained permission from the Costa Rican Park Service (SINAC, Área de Conservación Arenal Tempisque) to conduct research in the Lomas Barbudal Biological Reserve, as well as consent from private landowners who granted us access to their properties, including Hacienda Pelón, Brin d'Amor, and the community of San Ramón de Bagaces. The study was purely observational: no animals were captured or handled, no invasive procedures were performed, and no samples were imported or exported. Research protocols were approved by UCLA's Animal Care Committee (protocol 2016–2022), and all required permits from SINAC and MINAE (the Costa Rican agencies overseeing wildlife research) were secured and renewed every six months. The most recent authorizations include scientific passport #1012-2024-ACAT and permit Resolución #M-P-SINAC-PNI-ACAT-0010-2024. All procedures complied with the Animal Behavior Society's Guidelines for the Use of Animals in Research (https://doi.org/10.1016/S0003-3472(23)00317-2).
Disturbance	Our study was strictly noninvasive: animals were not captured, handled, or provisioned, and observers did not interact directly with them. The primary potential disturbance arises from habituation of capuchins to researcher presence, which may alter aspects of their behavior and reduce interactions with predators or competitors. This is an unavoidable outcome of all individual-based longitudinal studies on wild primates.

Reporting for specific materials, systems and methods

We require information from authors about some types of materials, experimental systems and methods used in many studies. Here, indicate whether each material, system or method listed is relevant to your study. If you are not sure if a list item applies to your research, read the appropriate section before selecting a response.

Materials & experimental systems

n/a	Involved in the study
☐	☐ Antibodies
☐	☐ Eukaryotic cell lines
☐	☐ Palaeontology and archaeology
☐	☒ Animals and other organisms
☐	☐ Clinical data
☐	☐ Dual use research of concern
☐	☐ Plants

Methods

n/a	Involved in the study
☐	☐ ChIP-seq
☐	☐ Flow cytometry
☐	☐ MRI-based neuroimaging

Antibodies

Antibodies used	N/A
Validation	N/A

Eukaryotic cell lines

Policy information about cell lines and Sex and Gender in Research

Cell line source(s)	N/A
Authentication	N/A
Mycoplasma contamination	N/A
Commonly misidentified lines (See ICLAC register)	N/A

Palaeontology and Archaeology

Specimen provenance	N/A
Specimen deposition	N/A
Dating methods	N/A

☐ Tick this box to confirm that the raw and calibrated dates are available in the paper or in Supplementary Information.

Ethics oversight	N/A

Note that full information on the approval of the study protocol must also be provided in the manuscript.

Animals and other research organisms

Policy information about studies involving animals; ARRIVE guidelines recommended for reporting animal research, and Sex and Gender in Research

Laboratory animals	N/A
Wild animals	This study did not involve capturing, handling, or killing any animals. All research was purely observational. The study species was the white-faced capuchin (Cebus imitator), with observed individuals ranging in age from 1 day to 34 years (and possibly older in cases where exact birth dates were unknown).
Reporting on sex	Findings apply to both female and male capuchins. In our models evaluating the effect of group size and seasonality on per capita fruit ingestion rate, we included a covariate indicating the sex of the individual in the model. Out of 336 individuals, 183 were male, 149 were female, and 4 were unknown. The data-set used for this analysis will be openly available after acceptance at the Dryad repository: https://doi.org/10.5061/dryad.612jm64j0.
Field-collected samples	No samples were collected that are relevant for this study.
Ethics oversight	Research protocols were approved by UCLA's Animal Care Committee (protocol 2016–2022), and all required permits from SINAC and MINAE (the Costa Rican agencies overseeing wildlife research) were secured and renewed every six months. The most recent authorizations include scientific passport #1012-2024-ACAT and permit Resolución #M-P-SINAC-PNI-ACAT-0010-2024.All procedures

complied with the Animal Behavior Society's Guidelines for the Use of Animals in Research (https://doi.org/10.1016/S0003-3472(23)00317-2).

Note that full information on the approval of the study protocol must also be provided in the manuscript.

Clinical data

Policy information about clinical studies

All manuscripts should comply with the ICMJE guidelines for publication of clinical research and a completed CONSORT checklist must be included with all submissions.

Clinical trial registration	N/A
Study protocol	N/A
Data collection	N/A
Outcomes	N/A

Dual use research of concern

Policy information about dual use research of concern

Hazards

Could the accidental, deliberate or reckless misuse of agents or technologies generated in the work, or the application of information presented in the manuscript, pose a threat to:

No	Yes	
☒	☐	Public health
☒	☐	National security
☒	☐	Crops and/or livestock
☒	☐	Ecosystems
☒	☐	Any other significant area

Experiments of concern

Does the work involve any of these experiments of concern:

No	Yes	
☒	☐	Demonstrate how to render a vaccine ineffective
☒	☐	Confer resistance to therapeutically useful antibiotics or antiviral agents
☒	☐	Enhance the virulence of a pathogen or render a nonpathogen virulent
☒	☐	Increase transmissibility of a pathogen
☒	☐	Alter the host range of a pathogen
☒	☐	Enable evasion of diagnostic/detection modalities
☒	☐	Enable the weaponization of a biological agent or toxin
☒	☐	Any other potentially harmful combination of experiments and agents

Plants

Seed stocks	N/A
Novel plant genotypes	N/A
Authentication	N/A

ChIP-seq

Data deposition

☐ Confirm that both raw and final processed data have been deposited in a public database such as GEO.

☐ Confirm that you have deposited or provided access to graph files (e.g. BED files) for the called peaks.

Data access links<br>*May remain private before publication.*	N/A
Files in database submission	N/A
Genome browser session<br>(e.g. UCSC)	N/A

Methodology

Replicates	N/A
Sequencing depth	N/A
Antibodies	N/A
Peak calling parameters	N/A
Data quality	N/A
Software	N/A

Flow Cytometry

Plots

Confirm that:

☐ The axis labels state the marker and fluorochrome used (e.g. CD4-FITC).

☐ The axis scales are clearly visible. Include numbers along axes only for bottom left plot of group (a 'group' is an analysis of identical markers).

☐ All plots are contour plots with outliers or pseudocolor plots.

☐ A numerical value for number of cells or percentage (with statistics) is provided.

Methodology

Sample preparation	N/A
Instrument	N/A
Software	N/A
Cell population abundance	N/A
Gating strategy	N/A

☐ Tick this box to confirm that a figure exemplifying the gating strategy is provided in the Supplementary Information.

Magnetic resonance imaging

Experimental design

Design type	N/A
Design specifications	N/A
Behavioral performance measures	N/A

Acquisition

Imaging type(s)	N/A
Field strength	N/A
Sequence & imaging parameters	N/A
Area of acquisition	N/A

Diffusion MRI ☐ Used ☐ Not used

Preprocessing

Preprocessing software	N/A
Normalization	N/A
Normalization template	N/A
Noise and artifact removal	N/A
Volume censoring	N/A

Statistical modeling & inference

Model type and settings	N/A
Effect(s) tested	N/A

Specify type of analysis: ☐ Whole brain ☐ ROI-based ☐ Both

Statistic type for inference (See Eklund et al. 2016)	N/A
Correction	N/A

Models & analysis

n/a	Involved in the study
☐	☐ Functional and/or effective connectivity
☐	☐ Graph analysis
☐	☐ Multivariate modeling or predictive analysis

Functional and/or effective connectivity	N/A
Graph analysis	N/A
Multivariate modeling and predictive analysis	N/A
